# Inhibition of penicillin-binding protein 2a (PBP2a) in methicillin resistant *Staphylococcus aureus* (MRSA) by combination of ampicillin and a bioactive fraction from *Duabanga grandiflora*

**DOI:** 10.1186/s12906-015-0699-z

**Published:** 2015-06-10

**Authors:** Carolina Santiago, Ee Leen Pang, Kuan-Hon Lim, Hwei-San Loh, Kang Nee Ting

**Affiliations:** Faculty of Science, University of Nottingham Malaysia Campus, Jalan Broga, 43500 Semenyih, Selangor Malaysia

**Keywords:** *Duabanga grandiflora*, MRSA, PBP2a, Synergistic activity, Antimicrobial

## Abstract

**Background:**

The inhibition of penicillin-binding protein 2a (PBP2a) is a promising solution in overcoming resistance of methicillin resistance *Staphylococcus aureus* (MRSA). A potential approach in achieving this is by combining natural product with currently available antibiotics to restore the activity as well as to amplify the therapeutic ability of the drugs. We studied inhibition effects of a bioactive fraction, F-10 (isolated from the leaves of *Duabanga grandiflora*) alone and in combination with a beta-lactam drug, ampicillin on MRSA growth and expression of PBP2a. Additionally, phytochemical analysis was conducted on F-10 to identify the classes of phytochemicals present.

**Methods:**

Fractionation of the ethyl acetate leaf extract was achieved by successive column chromatography which eventually led to isolation of an active fraction, F-10. Both extract and F-10 were analyzed for the presence of major classes of phytochemicals in addition to obtaining a high performance liquid chromatography (HPLC) profile to reveal the complexity of the fraction F-10. Broth microdilution method was employed to determine minimum inhibitory concentration (MIC) of the extract and fractions against MRSA. Evaluation of synergistic activity of the active fraction with ampicillin was determined using checkerboard methodand kinetic growth experiments. Effect of combination treatments on expression of PBP2a, a protein that confers resistance to beta-lactam antibiotics, was elucidated with the Western blot assay.

**Results:**

MIC of F-10 against MRSA was 750 mg/L which showed an improved activity by 4-fold compared to its crude extract (MIC = 3000 mg/L). Phytochemical analysis revealed occurrence of tannins, saponin, flavonoids, sterols, and glycosides in F10 fraction. In FIC index interpretation, the most synergistic activity was achieved for combinations of 1/64 × MIC ampicillin + 1/4 × MIC F-10. The combination also evidently inhibited MRSA growth in kinetic growth curve assay. As a result of this synergistic interaction, MIC of ampicillin against MRSA was reduced to 0.78 mg/L (64-fold) from initial value of 50 mg/L. Western blot analysis suggested inhibition of PBP2a in MRSA cultures grown in synergistic combination treatment in which no PBP2a band was expressed.

**Conclusions:**

The results demonstrated synergism between fraction F-10 of *D. grandiflora* with ampicillin in suppressing MRSA growth via PBP2a inhibition.

## Background

Resistance issue of MRSA continues to threaten the world population despite the availability of new antibiotics [1]. At present, MRSA infections are not only seen in hospital but also in community and livestock [2]. Prevalence of this infection underlies in its resistance mechanism. Primarily, acquisition of *mecA* gene confers resistance in *S. aureus* to methicillin. This gene encodes for production of a reduced-affinity penicillin-binding protein 2a (PBP2a) which was first discovered in 1981 [3]. The low binding affinity of PBP2a to beta-lactams enables peptidoglycan cell wall synthesis in MRSA despite the presence of lethal concentration of methicillin [4, 5]. Considering MRSA’s resistance, future quest of antimicrobials should be focused in finding solutions to inhibit production of PBP2a and restoring beta-lactams activity.

A plausible approach to treat infections caused by deleterious pathogens and to tackle its complex multi-drug resistance issues is by applying the concept of synergism [6]. Phytomedicine essentially, offers a new alternative for synergy treatment consisting combination of phytomedicine with commercially available antibiotics [7–9]. *Duabanga grandiflora* (Roxb. Ex DC) Walp, belongs to the *Lythraceae* family and it is indigenous to Eastern Himalayas [10, 11]. In India, the paste from the plant has been widely utilized to cure skin diseases, mainly eczema or atopic dermatitis (AD) [12]. Advancement in dermatological research indicated link between *S. aureus* infection and AD based on skin lesion caused by the bacteria and identification of *S. aureus* delta toxin in skin sample of AD patients [13, 14]. These findings show that ethnobotanical use of *D. grandiflora* in treating eczema may actually have relation to its ability to heal bacterial infections namely *S. aureus*.

In our earlier study we have shown that ethyl acetate extract from *D. grandiflora* possessed broad spectrum antimicrobial action including anti-staphylococcal activity [15, 16]. Based on this rationale, in the present study we investigated the synergistic effects of a bioactive fraction, F-10 from *D. grandiflora*,with ampicillin on MRSA growth. In order to better understand the molecular mechanism, effects of the combination treatment on the resistant protein, PBP2a was investigated. Chemical and HPLC analyses were conducted to identify the presence of major classes of compounds in the fraction as well as to gain a better insight into its complexity.

## Methods

### Plant material

*D. grandiflora* leaves were collected from a growing tree in Simpang Pulai, Pahang, Malaysia (GPS location: N04° 33.701’ E101° 11.685’) and identified by Dr. Christophe Wiart from the School of Pharmacy. Herbarium voucher specimens (herbarium code UNMC75) are deposited at the Herbarium of Faculty of Science, University of Nottingham Malaysia Campus. The dried and ground plant materials (2.1 kg – D*. grandiflora* leaves) was subjected to sequential extraction using *n*-hexane (He), followed by ethyl acetate (EA) and finally 95 % ethanol (EtOH). Extraction with each solvent was conducted by soaking the plant material in 10 L of the solvent (24 h × 3 times) at room temperature [16, 17].

### Isolation of F-10

The ethyl acetate extract of *D. grandiflora* leaves was fractionated by using vacuum liquid chromatography (silica gel). The solvent system used for elution was chloroform (CHCl_3_) in decreasing amount of hexane (He) or CHCl_3_ in increasing amount of methanol (MeOH), i.e., He/CHCl_3_ (1:1) → CHCl_3_ (100 %) → CHCl_3_/MeOH (3 %) → CHCl_3_/MeOH(5 %) → CHCl_3_/MeOH (7 %) → CHCl_3_/MeOH (10 %) → CHCl_3_/MeOH (15 %). The column was finally flushed with EtOH. Fraction F-10 eluted in solvent system CHCl_3_/MeOH (15 %).

### Microorganisms

Methicillin sensitive *S. aureus* ATCC 11632 (MSSA) was grown in tryptic soy broth (TSB) (Hi-Media, India) at 37 °C for 24 h with a shaking mode of 220 rpm. Aliquot from this suspension was streaked on tryptic soy agar (TSA) (Hi-Media, India) and incubated at 37 °C for another 24 h. Two to four single colonies from the TSA plate was inoculated in 10 ml of Muller Hinton broth (MHB) (Hi-Media, India) and allowed to grow at 37 °C until it reached exponential stage (2 × 10^8^ CFU/ml). The suspension then was used for broth microdilution assay. MRSA ATCC 43300 was grown with same steps except all the media used for its growth was supplemented with 2 % sodium chloride (NaCl) (Merck, Germany) and incubation temperature was 35 °C. Bacterial stocks were kept at −80 °C in TSB added with 10 % (vol/vol) glycerol (Sigma, USA).

### Test samples

The ethyl acetate crude extract of *D. grandiflora* and fraction F-10 were dissolved in dimethyl sulfoxide (DMSO) (Sigma, USA) at stock concentration of 1000 mg/L. The stock kept in −20 °C for experiments. Antibiotics for susceptibility testing prepared at 100 mg/L in sterile distilled water. Tested antibiotics were ampicilin (Amresco, USA), oxacillin (Discovery Fine Chemicals, UK) and methicillin (Sigma, USA).

### Determination of minimum inhibitory concentration (MIC)

Microbroth dilution method using a 96-well microtitier plate was used to determine MIC of crude extract *D. grandiflora*, fraction F-10 and control antibiotics against MRSA and MSSA. Antibiotics were tested with concentrations ranging from 0.19 to 100 mg/L and plant extract samples from 90 to 12,000 mg/L. Bacterial suspensions were prepared according to methods described above (section microorganism). Bacterial broth cultures were diluted to correspond to final inoculums of 5 × 10^5^ CFU/ml upon inoculation into each well containing two-fold serial dilution of test samples. This experiment was done according to guidelines from Clinical and Laboratory Standards Institute (CLSI) 2007 [18] with recommendations adapted from Cos et al. [19].

### Fractional inhibitory concentration (FIC) index interpretation- Checkerboard method

This experiment was conducted according to a previously described checkerboard method [20]. MRSA cultures were grown in presence of the following sub-inhibitory concentrations of F-10; 1/4 × MIC (188 mg/L), 1/8 × MIC (94 mg/L),1/16 × MIC (47 mg/L), and 1/32 × MIC (23 mg/L) in combination with sub-inhibitory concentrations of ampicillin ranging from 1/2 × MIC to 1/64 × MIC (25 mg/L to 0.78 mg/L). This experiment was conducted on a 96-well microtiter plate with test samples and bacterial suspension prepared in same manner as for MIC determination except wells now contained combined test samples (F-10 and ampicillin).

FIC index for the combination treatments were calculated. The formula used was:

$$ \mathrm{F}\mathrm{I}\mathrm{C}\ \mathrm{ampicillin} = \mathrm{MIC}\ \mathrm{of}\ \mathrm{ampicillin}\ \mathrm{in}\ \mathrm{combination} \div \mathrm{MIC}\ \mathrm{of}\ \mathrm{ampicillin}\ \mathrm{alone} $$

$$ \mathrm{F}\mathrm{I}\mathrm{C}\ \mathrm{plant}\ \mathrm{extract} = \mathrm{MIC}\ \mathrm{of}\ \mathrm{plant}\ \mathrm{extract}\ \mathrm{in}\ \mathrm{combination} \div \mathrm{MIC}\ \mathrm{of}\ \mathrm{plant}\ \mathrm{extract}\ \mathrm{alone} $$

$$ \mathrm{F}\mathrm{I}\mathrm{C}\ \mathrm{index} = \mathrm{F}\mathrm{I}\mathrm{C}\ \mathrm{of}\ \mathrm{ampicillin} + \mathrm{F}\mathrm{I}\mathrm{C}\ \mathrm{of}\ \mathrm{plant}\ \mathrm{extract} $$

The combination was defined as synergy if the FIC index is ≤ 0.5, indifference was defined as when FIC index is > 0.5 but ≤ 4.0, and antagonism was defined as when FIC index is > 4.0 [20]. The combination of two test samples; ampicillin and fraction F-10 was graphically described by a Cartesian diagram using isobole method for combinations that lowered MIC ampicillin. A straight line indicates no interaction and a concave isobole shows synergism between the tested components [21].

### Kinetic growth curves assay

The experiment was conducted for combination which lowered the MIC of ampicillin for MRSA from 50 mg/L to 0.78 mg/L (1/64 of the original MIC). The test samples and bacterial inoculums were prepared as the same for FIC interpretation on a 96- well microtiter plate. Cell growth was monitored by reading optical density (OD) values at 600 nm at indicated time points for 24 h. Reading was monitored by using Varioskan flash multimode reader (Thermo Scientific, USA).

### Western blotting analysis

MRSA culture was grown in combination treatment consisting of 1/64 × MIC ampicillin + 1/4 × MIC F-10 until late exponential phase (24 h). Bacterial protein was extracted by preparing the lysates in an extraction buffer containing Tris and EDTA (Thermo Scientific, USA). Following 15-min of centrifugation at 18,500 g, the pellets were obtained as the insoluble protein extracts which was harvested in elution buffer containing Tris, urea and sodium dihydrogen phosphate. Extracted protein (3 mg/L) was subjected to sodium dodecyl sulfate (SDS) - polyacrylamide (12 %) gel electrophoresis ran at 120 V, and then transferred to BioTrace™ NT nitrocellulose transfer membrane (Pall, USA). The production of PBP2a from MRSA was detected by probing the membranes with mouse anti-PBP2a primary antibody (Denka Seiken, Japan) and anti-glyceraldehyde 3-phosphate dehydrogenase (GAPDH) (Thermo Scientific, USA) with dilution factor of 1:10000. The same membranes were re-probed with anti-mouse horseradish peroxidase-linked secondary antibody (Abcam, UK) diluted to 1:10000 to facilitate colorimetric detection with 3, 3’, 5, 5’-tetramethylbenzidine (TMB) substrate (Nacalai Tesque, Japan). Assay response was recorded using GS-800™ calibrated densitometer (Bio-Rad, USA). Results were recorded based on visual band intensity.

### Phytochemical analysis

Phytochemical analysis for the ethyl acetate crude extract of *D. grandiflora* leaves and fraction F-10 was carried out according to methods described by Jones and Kinghorn [22].

### High performance liquid chromatography (HPLC) analysis

An aliquot of fraction F-10 (40 μl of 10 mg/ml) was analyzed by reverse phase HPLC (C_18_) using the following gradient solvent system: 2 min at 10 % acetonitrile (ACN)/miliQ water (H_2_O); a linear gradient to 75 % ACN/H_2_O over 12 min; isocratic at 75 % for 10 min; a linear gradient to 100 % ACN for 2 min; isocratic at 100 % ACN for 4 min. HPLC was performed on a Varian 940-LC system using a reversed phase analytical column (Pursuit XRs C18, 4.6 × 150 mm, 5 μm) with photodiode array (PDA) detection at 254 nm.

## Results

### Anti-MRSA activities of *D. grandiflora* leaf extract and fraction F-10

The MIC values for MRSA are higher than MSSA for the tested antibiotics confirming the resistance of the strain used in this study. *D. grandiflora* leaf extract showed a moderate antimicrobial activity against MRSA and MSSA with same MIC value. The MIC value for fraction F-10 however showed a 4-fold reduction indicating an increased antimicrobial activity against MRSA and MSSA. Table 1 shows the MIC results for tested antibiotics, *D. grandiflora* extract and F-10.

**Table 1:** Minimum inhibitory concentration (MIC) of test samples for MRSA ATCC 43300 and MSSA ATCC 11632. Values represent triplicates of three independent experiments

	MIC (mg/L)	
Test samples	MRSA ATCC 43300	MSSA ATCC 11632
Ampicillin	50	6.25
Oxacillin	20	2.50
Methicillin	10	0.31
Leaf ethyl acetate extract *D. grandiflora*	3000	3000
Fraction F-10	750	750

### Synergistic effects of ampicillin and fraction F-10 against MRSA FIC index

In FIC index interpretation, 10 out of 24 tested combination treatments indicated synergism (results not shown). Table 2 shows the new MIC values of ampicillin against MRSA achieved by combination treatments for four most synergistic combinations along with the corresponding FIC index values. It is noteworthy that the presence of F-10 remarkably reduced the MIC of ampicillin against MRSA. The most notable change in the MIC of ampicillin was observed when 1/4 × MIC of F-10 was added, where the MIC was reduced by 64-fold from 50 mg/L to 0.78 mg/L.

**Table 2:** FIC indices combination of fraction F-10 of *D. grandiflora* leaf extract with ampicillin against MRSA ATCC 43300. Values represent triplicates of three independent experiments

MIC of F-10 (mg/L)	MIC of ampicillin in combination (mg/L)	^a^FIC index
0	50	-
1/4 × MIC (188)	0.78	0.25
1/8 × MIC (94)	3.13	0.18
1/16 × MIC (47)	12.5	0.31
1/32 × MIC (23)	12.5	0.28

^a^FIC index; synergism ≤ 0.5, indifference >0.5 but ≤ 4.0, and antagonism > 4

**Fig. 1:**
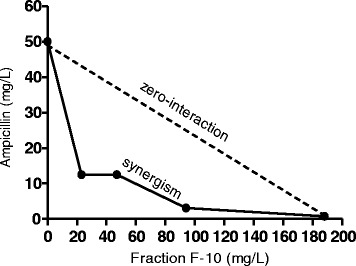
Isobole MIC curve. Isobole MIC curve for MRSA ATCC 43300 describing synergism between ampicillin and fraction F-10 of *D. grandiflora* leaf extract at sub-inhibitory concentrations

The appreciable synergistic effects observed for the combination 1/64 × MIC ampicillin + 1/4 × MIC F-10 is further supported by the isobole concave curve for the MIC of the two components, ampicillin and F-10 (Fig. 1).

### Effects of ampicillin and F-10 alone and in combination on MRSA growth curves

**Fig. 2:**
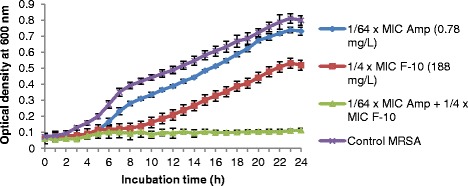
Growth curves of MRSA ATCC 43300. MRSA cultures were grown in sub-inhibitory concentrations of fraction F-10 alone, ampicillin alone and in combination. Cell growth was measured by using optical density at 600 nm at indicated time points. The curves represent triplicates of three independent experiments. Error bars show the standard deviation ( 1/64 × MIC Amp, 1/4 × MIC F-10,  combination of Amp + F-10,  control MRSA, Amp = ampicillin, MIC = minimum inhibitory concentration)

Kinetic growth curves of combinations of ampicillin and fraction F-10 demonstrated synergistic effects in inhibiting growth of MRSA over a period of 24 h (Fig. 2). The presence of F-10 alone at sub-inhibitory concentrations (1/4 × MIC) suppressed the bacterial growth up to 50 % compared to control MRSA. Ampicillin at sub-inhibitory concentration (1/64 × MIC) suppressed the growth of MRSA by 8 % only after 24-h of incubation. Nevertheless, in combination treatment, the growth of MRSA was markedly inhibited.

### Inhibition of PBP2a expression in MRSA by combination of ampicillin with F-10

**Fig. 3:**
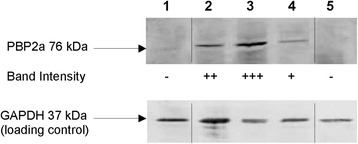
Inhibition of PBP2a expression in MRSA ATCC 43300. Lane: 1 - control MSSA ATCC 11632, 2 - control MRSA ATCC 43300, 3 - ampicillin 0.78 mg/L, 4 - fraction F-10 188 mg/L, 5 - ampicillin 0.78 mg/L + fraction F-10 188 mg/L. Scoring of visual band intensity; − absence of band, + presence of band, ++ presence of strong band, +++ presence of very strong band (black vertical lines in between the gel lanes indicate the image has been juxtaposed but within the same gel)

The expression of PBP2a in MRSA in treatment of 1/64 × MIC ampicillin + 1/4 × MIC F-10 was studied. The controls include untreated MRSA, untreated MSSA and MRSA grown in only one test sample (ampicillin or F-10) at sub-inhibitory concentrations that were same as in the combination treatment. Figure 3 depicts expression of PBP2a in each treatment. MSSA did not express any PBP2a band and a clear protein band (76 kDa) was seen in MRSA cultures. The intensity of PBP2a expression in MRSA cultures that were exposed to 1/64 x MIC ampicillin was higher compared to untreated MRSA (See Fig. 3, lane 3 and lane 2, respectively). MRSA culture treated with F-10 alone appeared to have attenudated PBP2a expression compared to control MRSA (see lane 4). In combination treatment, presence of F-10 and ampicillin (both at sub-inhibitory level), totally inhibited the expression of PBP2a. Control GAPDH was expressed in all controls and treatment.

### Classes of phytochemicals and HPLC analysis of fraction F-10

Phytochemical evaluation of the crude extract indicated the presence tannins, alkaloids, flavonoids, saponins, sterols and glycosides, while fraction F-10 contain similar classes of compounds, except alkaloid (Table 3).

**Table 3:** Qualitative analysis of phytochemical contents of *D. grandiflora* leaves extract and fraction F-10

Phytochemicals	*D. grandiflora* leaves extract	F-10
Tannins	+ +	+
Alkaloids	+	-
Flavonoids	+	+
Saponins	+	+
Sterols	+ +	+
Glycosides	+ +	+ +

Results represent triplicates of an independent experiment (+ present, + + moderate amount, + + + appreciable amount, −absence)

**Fig. 4:**
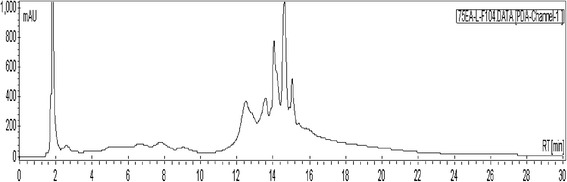
HPLC chromatogram of fraction F-10 with PDA detection. Reverse phase HPLC analysis of fraction F-10 (40 μl of 10 mg/ml) showing presence of five broad chromatographic peaks detected at 254 nm

HPLC chromatogram (Fig. 4) of fraction F-10 revealed five broad chromatographic peaks at 254 nm. These peaks appeared when the mobile phase composition was 75 % ACN/H_2_O, taking place between 12 to 16 min. The chromatogram indicated severe overlapping of individual compound peak due to incomplete separation, which suggested that F-10 comprised a complex mixture of phytochemicals and this is consistent with the phytochemical analysis.

## Discussion

Medicinal use of *D. grandiflora* is to treat ailments associated with skin diseases [12] which may worsen with bacterial and viral infection due the patient’s susceptibility [23]. Consistently, MIC evaluation indicated that the crude ethyl acetate extract and fraction F-10 possess antibacterial properties against MRSA and MSSA based on their antimicrobial activities. Deduction from the MIC and FIC index evaluations coupled with results from the kinetic growth assay concluded that components in F-10 act synergistically with ampicillin in inhibiting MRSA’s growth. This was evidently demonstrated by reduction of MIC ampicillin from 50 mg/L to 0.78 mg/L in the presence of 1/4 × MIC of F-10.

Prompted by these results, we investigated the combination’s mechanisms of action on MRSA and found it is linked to PBP2a inhibition. Shimizu et al. [24] reported that corilagin from *Arctostaphylos uva-ursi* was able to inhibit PBP2a production and subsequently increased the potency of beta-lactam antibiotics. Based on the Western blot analysis, we confirmed that combination of F-10 with ampicillin (both at sub-inhibitory concentration) totally suppressed the expression of PBP2a in MRSA. The noteworthy observation here is F-10 alone could only attenuate PBP2a expression in MRSA and by combining this fraction with ampicillin, a complete inhibition on PBP2a was achieved. We postulated that F-10 interferes with the regulatory genes involved in the expression of *mecA* that encodes for PBP2a expression in MRSA [25]. This gene’s transcription can be controlled by the *mecR1-mecI-mecR2* regulatory gene. It was reported that this very unique three-component arrangement, consisting of a transcriptional repressor (*mecI*), a sensor-inducer (*mecR1*) and an anti-repressor (*mecR2*), is vital for full expression of the *mecA* gene [26–28]*.* In brief, exposure of beta-lactams to MRSA stimulates *mecR1-mecI-mecR2* genes of *mecA* regulatory locus, which leads to robust induction of *mecA* transcription. As a consequence, unique modulation of proteolytic cleavage series takes place, eventually causing complete expression of beta-lactam resistance such as PBP2a production [26]. Hence, intrusion of F-10 with any of these regulatory genes might interrupt the three-component arrangement system, consequently preventing synthesis of PBP2a or full induction of *mecA* that is vital for resistance expression. Nevertheless, the possibility that the antibacterial effects of F-10 is caused by several mechanisms cannot be ruled out since this fraction essentially contains a cocktail of naturally occurring compounds.

Phytochemical analysis showed occurrence of tannins, flavonoids, saponins, sterols, and glycosides in F-10. Same phytochemicals were detected in *D. grandiflora* extracts reported in a different study that demonstrated anti-staphylococcal activities [15]. The phytochemical analysis also provided insight into possible mechanisms of action. Flavonoids and tannins found in F10 have been shown to be toxic to microorganism due sites and numbers of hydroxyl group that has non nonspecific interactions with the protein or able to cause enzyme inhibition [29, 30]. Since PBPs are group of protein enzymes [31], these phytochemicals could form non-specific interaction with them. Interaction as this may affect the bacterial cell biosynthesis process eventually leading to MRSA growth inhibition as demonstrated in our synergy growth curve experiment. Furthermore, it is likely that the same non-specific interaction may have also caused inhibition of PBP2a expression in our study. Besides that sterols and glycosides were also found in F-10. Plant sterols were proven to have antibacterial properties [32], and several glycoside compounds were reported to exhibit antimicrobial activities against MRSA [33]. It appears that a number of possible phytochemicals detected in F-10 could contribute to the anti-MRSA activity.

Our study indicates that F-10 has anti-MRSA properties and resulted in greater inhibitory activity when used with ampicillin in the in-vitro assays. Synergism of antimicrobial effect in this study is linked to inhibition of PBP2a expression. As such F-10 is potentially a good candidate for development of new treatment regime for MRSA. Subsequent experiments are crucial to study the pharmacokinetics properties of the fraction and its interaction with ampicillin in an in vivo model. Besides, the interactions of F-10 with the regulatory genes, *mecI-mecR1-mecR2* and their resultant proteins leading to the inhibition phenomenon remained to be solved and require additional investigations.

## Conclusions

Our data revealed that a bioactive fraction F-10 isolated from *D. grandiflora* possesses antimicrobial activity. Anti-MRSA evaluation showed that this fraction works synergistically with ampicillin in hindering growth of the bacteria. The ability of F10 to attenuate the production of PBP2a is postulated to result in the restoration of the susceptibility of MRSA to ampicillin.
